# Supporting the right ventricle in postcardiotomy renal dysfunction: A case series

**DOI:** 10.1002/ccr3.7695

**Published:** 2023-07-17

**Authors:** Mami Sow, Benjamin D. Seadler, Sonal R. Chandratre, Abhilash Koratala, Samuel F. Carlson, Lyle D. Joyce, Takushi Kohmoto, Lucian A. Durham, David L. Joyce

**Affiliations:** ^1^ Medical College of Wisconsin Milwaukee Wisconsin USA; ^2^ Division of Cardiothoracic Surgery Froedtert & Medical College of Wisconsin Milwaukee Wisconsin USA; ^3^ Department of Pediatric Endocrinology Aspirus Health Stevens Point Wisconsin USA; ^4^ Division of Nephrology Froedtert & Medical College of Wisconsin Milwaukee Wisconsin USA; ^5^ Department of Surgery University of Iowa Iowa Iowa USA

**Keywords:** acute kidney injury, ECMO, right ventricular assist device, right ventricular failure

## Abstract

Postcardiotomy RV dysfunction is an under‐recognized cause of acute kidney injury (AKI). Insertion of a percutaneous right ventricular assist device (RVAD) reduces central venous hypertension and congestive nephropathy by augmenting cardiac output. In selected patients, percutaneous RVAD insertion may improve renal function and obviate the need for long‐term dialysis.

## 1 INTRODUCTION

Cardiorenal syndrome is a cyclical bidirectional disorder of the heart and the kidneys where dysfunction in one system induces and augments dysfunction in the other. In the setting of right heart failure, rising CVP is a central cause of renal dysfunction. Increasing backpressure leads to an increase in renal venous pressure, perpetuating AKI via a reduction in glomerular filtration rate (GFR).^1^ This phenomenon has been termed congestive nephropathy; a reversible pathology if addressed early in its course.^2^ The rates of in‐hospital and one‐year morbidity and mortality are significantly increased for patients with postcardiotomy cardiorenal syndrome. Early identification and amelioration of kidney injury has profound implications on the trajectory of a patient's life course after a major cardiac operation.

The following is a case series of three patients with postcardiotomy AKI who underwent percutaneous RVAD placement and had subsequent return of meaningful renal function while avoiding long‐term renal replacement therapy.

## 2 METHODS

We present the cases of three patients who underwent treatment for ACC/AHA Stage D heart failure with reduced ejection fraction: two with left ventricular assist device (LVAD) insertion and one with orthotopic heart transplant. Postoperatively, these patients developed renal dysfunction with clinical findings of RVF. In each case, percutaneous RVAD insertion resulted in renal recovery as evidenced by improvement in serum creatinine and urine output. The patients were weaned from right‐sided cardiac support and the RVADs were able to be removed. None of the three patients required long‐term renal replacement therapy.

## 3 RESULTS

### 3.1 Case 1

A 73‐year‐old man with a history of coronary artery disease, hyperlipidemia, hypertension, and heart failure with reduced ejection fraction secondary to ischemic heart disease who was admitted to the Cardiovascular Intensive Care Unit (CVICU). He was listed for cardiac transplantation as Status 1 due to inability to wean from percutaneous RVAD and LVAD support. The patient underwent successful orthotopic heart transplantation. On post‐transplant day 1, he had adequate filling pressures with a CVP of 10 mmHg (goal CVP of less than 12 mmHg), creatinine increase from 0.7 to 1.2 mg/dL, and a notable decline in urine output from 1.2 cc/kg/h the day prior to less than 0.5 cc/kg/h (reference range: 1–2 cc/kg/h). The patient's vasopressor requirements increased throughout the day, his pulmonary artery pulsatility index (PaPi) decreased to 0.83 mmHg (goal of >1), and his CVP rose to 20 mmHg prompting a return to the operating room for further exploration.

**FIGURE 1:**
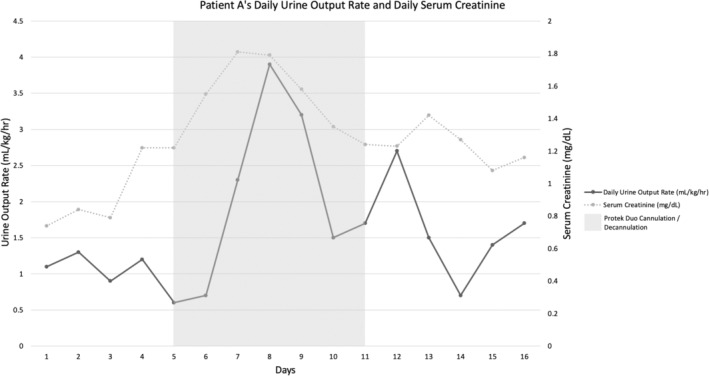
Daily urine output and serum creatinine in relation to insertion and removal of percutaneous RVAD for Patient A.

Intraoperatively, it was found that the dysfunction was partially related to oversizing and edema of the graft. Ongoing RV dysfunction prompted insertion of a percutaneous RVAD via the right internal jugular vein. Shortly after returning from the operating room, the patient had continued low urine output. The RVAD flows were initially ~5 L at 4000 RPM in the operating room but decreased to ~3.5 L in the CVICU. Urine output and RVAD flows did not improve with additional resuscitation. The patient began exhibiting clinical signs of superior vena cava syndrome, and he was promptly returned to the operating room for cannulation of the subclavian and femoral veins to reduce central venous congestion. Within 24 hours, his urine output increased significantly to 2.3 cc/kg/h and 3.9 cc/kg/h by 48 h. The RVAD was weaned, and on postplacement day 6, it was removed. By this time, his creatinine had decreased to 1.24 mg/dL and his urine output remained robust at 1.7 cc/kg/h (Figure 1). He had a prolonged course in the ICU with chronic hypoxic respiratory failure in the setting of COVID‐19 infection necessitating tracheostomy. After several months of recovery and inpatient rehabilitation, he was discharged home without the need for any form of renal replacement therapy.

### 3.2 Case 2

A 72‐year‐old man with American College of Cardiology/American Heart Association Stage D HFrEF due to mixed ischemic and nonischemic cardiomyopathy was admitted for scheduled LVAD insertion. At the time of admission, he had been inotrope‐dependent for 8 months. The patient's admission creatinine was 1.45 mg/dL, consistent with stage III chronic kidney disease. Immediately prior to surgery, the patient underwent right heart catheterization, which revealed a PaPi of 9.3 and CVP of 3 mmHg. The patient had an LVAD placed the following day, which was complicated by coagulopathy necessitating delayed sternal closure. On POD 2, his chest was closed. Intraoperative transesophageal echocardiogram (TEE) revealed adequate RV function and PaPi of 1.5.

**FIGURE 2:**
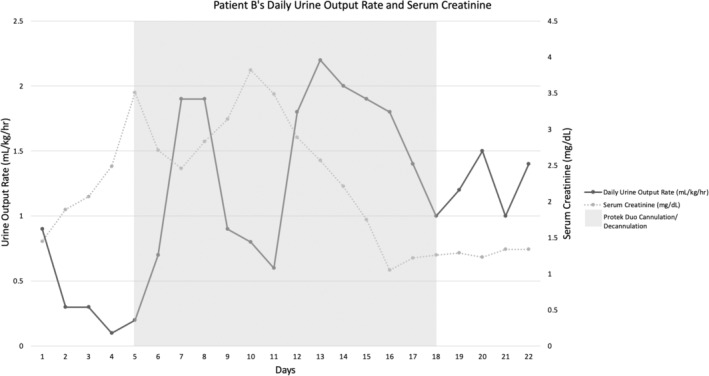
Daily urine output and serum creatinine in relation to insertion and removal of percutaneous RVAD for Patient B.

By POD 10, the patient had been weaned to a single inotrope and was being diuresed. It was noted that although his PaPi scores were previously consistently above 1.0, they had dropped to 0.94 with a rising CVP to 16 mmHg. On POD 12, the patient's serum creatinine had risen from 1.45 mg/dL to 1.89 mg/dL. Left ventricular assist device speed was increased for additional left ventricular offloading. The patient's creatinine continued to rise, up to 3.51 mg/dL on POD 15. Urine output dropped precipitously to 0.1 cc/kg/h during this time. The decreased PaPi and worsening kidney injury were concerning for ongoing RV dysfunction. The decision was made to proceed with percutaneous RVAD insertion via the right internal jugular vein. He was concurrently started on continuous renal replacement therapy (CRRT) due to persistent periprocedural hyperkalemia. Overnight, the patient's urine output improved to 0.7 cc/kg/h while his creatinine improved from 3.51 mg/dL to 2.71 mg/dL. He was weaned from CRRT within 24 h. By 48 h, his creatinine decreased to 2.29 mg/dL and urine output was 1.9 cc/kg/h. The RVAD remained in place for 13 days, and by decannulation, the patient had a urine output of 1 cc/kg/h, a serum creatinine of 1.26 mg/dL, and a CVP of 10 mmHg (Figure 2). The patient continued to recover and was discharged home several weeks later.

### 3.3 Case 3

**FIGURE 3:**
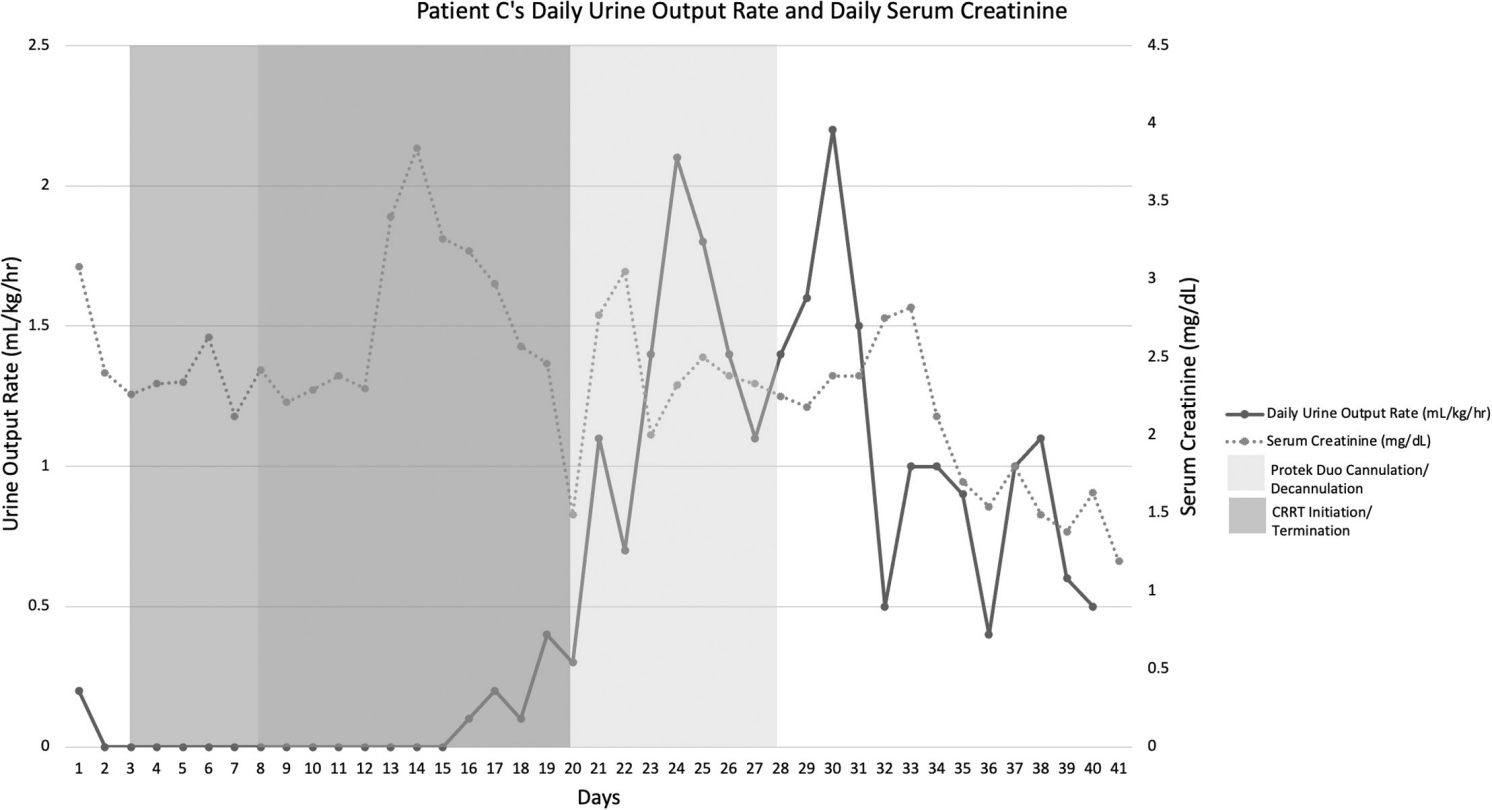
Daily urine output and serum creatinine in relation to insertion and removal of percutaneous RVAD and CRRT for Patient C.

A 48‐year‐old man with a history of ACC/AHA Stage D heart failure with reduced ejection fraction secondary to nonischemic cardiomyopathy was admitted for planned LVAD exchange secondary to pump thrombosis resulting in recent ischemic cerebrovascular accident. Previous right heart catheterization revealed PaPi of 2.8 and a CVP of 5 mmHg. Postoperatively, his condition stabilized and his vasopressor requirement decreased over the ensuing days. Despite adequate UOP, his serum creatinine began to rise from 1.2 to 2.5 mg/dL, and he developed AKI. His urine output initially responded to diuretics, but by POD 12, he had persistent renal dysfunction and became anuric, requiring initiation of CRRT. By POD 21, he had persistently elevated CVP and low PaPi, despite having adequate CO, cardiac index (CI), and mean arterial pressure (MAP) without vasopressor assistance. The decision was made to insert a percutaneous RVAD to decompress the right ventricle and attempt to alleviate his presumed cardiorenal syndrome. Right ventricular assist device insertion allowed for significantly increased LVAD speeds improving CO. His renal function slowly improved (Figure 3), and the RVAD was weaned. On POD 26, he was transitioned to prolonged intermittent renal replacement therapy and then to intermittent hemodialysis on POD 33. His last session of hemodialysis was on POD 35. The percutaneous RVAD was removed POD 40, he was transferred to the floor on POD 44, and discharged to a rehabilitation facility on POD 53. There are no outpatient hemodialysis centers in this geographic region that will accept patients with an LVAD; it was critically important that he no longer require renal replacement therapy to be discharged from the hospital.

## 4 DISCUSSION

Presented is a case series of three patients who developed AKI following LVAD insertion or heart transplant and who were able to avoid long‐term dialysis with temporary percutaneous RVAD insertion. Acute kidney injury remains one of the most common complications following cardiac surgery with an incidence of approximately 40%.^3^ When looking at cardiac transplantation specifically, the incidence rises as high as 60% with 10% of patients requiring renal replacement therapy postoperatively.^4^ The development of AKI is associated with a higher risk of subsequent morbidity and mortality. Rates of prolonged ventilator dependence, respiratory failure, right ventricular failure, 30‐day mortality, and 1‐year mortality are significantly increased.^5^ A smaller percentage of patients continue to require renal replacement therapy after discharge. Long‐term hemodialysis is associated with diminished quality of life scores in both functional and emotional domains.^6^

As previously stated, LVAD implantation and cardiac transplantation carry a particularly increased risk of postoperative kidney injury. Anywhere from 5.7% to 19.7% of these patients require some form of renal replacement therapy, which is associated with worse long‐term prognosis, morbidity, and mortality.^7^, ^8^ A recent study by Silver et al highlights the devastating consequences of this prevalent complication.^9^ In their analysis of the National Inpatient Sample, the in‐hospital mortality in patients that developed AKI was four times higher than those that did not develop AKI and 10 times higher in patients that ultimately required dialysis. Transitioning to outpatient dialysis can be logistically arduous in patients with complex cardiac conditions. Given the high level of care associated with these patients, it can prove difficult to find an outpatient dialysis center willing to accept patients who have undergone transplant or LVAD insertion.

There are limited interventions that serve to reliably prevent AKI following cardiac surgery. Intensivists use a variety of physical examination findings, invasive monitoring catheter data, laboratory testing, and point‐of‐care ultrasound imaging to attempt to assess fluid status and intravascular volume. Goals include maintaining low CVP (usually less than 12 mmHg) and adequate MAPs to optimize end‐organ perfusion. These are accomplished through judicious fluid resuscitation, hemodynamic support including inotropes and vasopressors, and diuresis to minimize vascular congestion. Once renal dysfunction occurs, it can potentiate the decline of other inter‐related organ systems. These three cases highlight the potential impact of early RVAD support in combating this complication. Despite the well‐described relationship of renal failure in the setting of RV dysfunction, there are little published data regarding the impact of temporary RV support in preventing permanent kidney dysfunction. Moayedifar et al. published a retrospective single‐institution study in 2021, which assessed the effect of temporary RVAD implantation in terminal heart failure patients who underwent LVAD implantation.^10^ Patients who had impaired renal function at the time of RVAD insertion had significant improvement in renal function. Additional research is needed beyond patients undergoing LVAD insertion to better understand this phenomenon.

Right ventricular failure remains a significant cause of morbidity and mortality following cardiac surgery. There are several factors that make the right ventricle more susceptible to failure compared with the left ventricle. The right ventricle is anatomically the more anterior of the two ventricles and thus is more susceptible to myocyte damage from thermal injury during cardiopulmonary bypass. In addition, retrograde cardioplegia may not adequately perfuse the myocardium of the right ventricle. The presence of right coronary artery disease can limit adequate protection during antegrade cardioplegia.^11^ The resulting RVF, if not addressed, can lead to dysfunction in associated organ systems. Renal dysfunction is both an indicator and potentiator of this decline. In the past, acute RVF has typically required surgical implantation of an RVAD. Several prior studies have reported high rates of associated morbidity, with published rates of weaning from surgically placed RVADs in the realm of 49%–59%.^12^ Percutaneous RVAD support has become increasingly adopted in cases of perioperative RVF. A single‐center retrospective study in 2020 demonstrated a 94.4% success rate of weaning off percutaneous RVAD postcardiotomy, but also a 90% survival rate, thus underscoring the benefit on patient outcomes.^13^ Additional studies have renumerated the benefits of percutaneous RVADs including avoidance of sternotomy, decreased periprocedural sedation, decreased postoperative pain, decreased duration of mechanical ventilation, and earlier ambulation.^14^, ^15^

This case series demonstrates a potential relationship between percutaneous RVAD insertion and amelioration of renal dysfunction in patients undergoing durable LVAD implantation or heart transplantation. We are cautiously encouraged by the results, which we feel highlight the importance of recognizing subtle clinical signs of RVF and initiating early right ventricular mechanical support. A prospective study is currently planned to assess the rate of renal recovery in patients who undergo percutaneous RVAD insertion to investigate its ability to potentially abate the need for renal replacement therapy.

## AUTHOR CONTRIBUTIONS

**Mami Sow:** Conceptualization; data curation; investigation; methodology; writing – original draft; writing – review and editing. **Benjamin D Seadler:** Investigation; methodology; writing – original draft; writing – review and editing. **Sonal R. Chandratre:** Conceptualization; writing – review and editing. **Abhilash Koratala:** Conceptualization; writing – review and editing. **Samuel F. Carlson:** Conceptualization; data curation; investigation; writing – review and editing. **Lyle D. Joyce:** Writing – review and editing. **Takushi Kohmoto:** Writing – review and editing. **Lucian A Durham:** Writing – review and editing. **David L. Joyce:** Conceptualization; supervision; writing – review and editing.

## FUNDING INFORMATION

None.

## CONFLICT OF INTEREST STATEMENT

David L. Joyce MD, MBA, is a member of the steering committee for the THEME registry. There are no other conflicts of interest to disclose.

## CONSENT STATEMENT

Written informed consent was obtained from the patients to publish this report in accordance with the journal's patient consent policy.

## Data Availability

Data available on request from the authors.
